# The left ventricular assist device ‘skeleton man’: case report—simple tools for skeletal muscle evaluation and very early aerobic/resistance/inspiratory training in cardiac cachexia

**DOI:** 10.1093/ehjcr/ytae401

**Published:** 2024-08-07

**Authors:** Ioannis D Laoutaris, Aggeliki Gkouziouta, Michael J Bonios, George Katelouzos, Nektarios Kogerakis, Themistocles Chamogeorgakis, Stamatis Adamopoulos

**Affiliations:** Cardiac Rehabilitation Department, Onassis Cardiac Surgery Center, 356 Sygrou Blvd, 17674 Athens, Greece; Heart Failure, Mechanical Support and Transplant Unit, Onassis Cardiac Surgery Center, 356 Sygrou Blvd, 17674 Athens, Greece; Heart Failure, Mechanical Support and Transplant Unit, Onassis Cardiac Surgery Center, 356 Sygrou Blvd, 17674 Athens, Greece; Heart Failure, Mechanical Support and Transplant Unit, Onassis Cardiac Surgery Center, 356 Sygrou Blvd, 17674 Athens, Greece; Cardiac Rehabilitation Department, Onassis Cardiac Surgery Center, 356 Sygrou Blvd, 17674 Athens, Greece; Heart Failure, Mechanical Support and Transplant Unit, Onassis Cardiac Surgery Center, 356 Sygrou Blvd, 17674 Athens, Greece; Heart Failure, Mechanical Support and Transplant Unit, Onassis Cardiac Surgery Center, 356 Sygrou Blvd, 17674 Athens, Greece; Heart Failure, Mechanical Support and Transplant Unit, Onassis Cardiac Surgery Center, 356 Sygrou Blvd, 17674 Athens, Greece

**Keywords:** Case report, LVAD, Heart failure, ARIS training, Exercise, Frailty, Skeletal muscle, Cachexia

## Abstract

**Background:**

Skeletal muscle wasting (SMW) is highly prevalent in patients with heart failure (HF) at left ventricular assist device (LVAD) implantation and is associated with morbidity and mortality. At the same time, SMW is clinically under-recognized, while exercise training (ET) studies in weak LVAD patients are lacking.

**Case summary:**

A 60-year-old man with advanced HF, SMW, cardiac cachexia, and frailty was confined in bed for 6 months initially supported with intravenous inotropes and subsequently with an intra-aortic balloon pump. His frailty was recognized as an LVAD-responsive frailty, and patient was successfully implanted with a HeartWare (Medtronic). Post-surgery, patient was very weak, unable even to move in bed without assistance. We evaluated skeletal muscle using simple tools such as the Oxford scale, mid-thigh circumference, hand-held dynamometry, and maximum inspiratory pressure. Physical performance was assessed with the sit to stand test, gait speed test, pedal bike timing, and the 6 min walk test. On top of routine physiotherapy, patient underwent an 8-week modified aerobic/resistance/inspiratory (ARIS) ET programme at moderate intensity and showed significant improvements in skeletal muscle mass and strength and physical and functional capacity.

**Discussion:**

We want to emphasize the importance of skeletal muscle evaluation at LVAD implantation and the feasibility and effectiveness of early ARIS training in very weak patients.

**Learning points**

- Objective assessment of skeletal muscle/physical performance/sarcopenia and cachexia status is warranted in all patients at left ventricular assist device (LVAD) implantation for individualized rehabilitation prescription.
- Left ventricular assist device implantation might be feasible in highly selected patients with cardiac cachexia.
- Modified aerobic/resistance/inspiratory exercise training, started as early as possible, may allow successful cardiac rehabilitation in highly selected LVAD recipients despite baseline cardiac cachexia.

## Introduction

Cardiac cachexia is a syndrome characterized by weight loss, anorexia, skeletal muscle wasting (SMW), and inflammation. It occurs in 10–20% of patients with heart failure (HF), recognized as an independent risk factor for mortality and is usually regarded as a *signum mali ominis*.^1–3^ Recent studies also showed that SMW is present in half of the HF patients at left ventricular assist device (LVAD) implantation and is associated with re-hospitalizations and mortality.^4–6^ In this case report, we present our approach to the physical recovery of a HF patient with SMW, cardiac cachexia, and frailty, very early post-LVAD implantation.

## Summary figure

**Figure:**
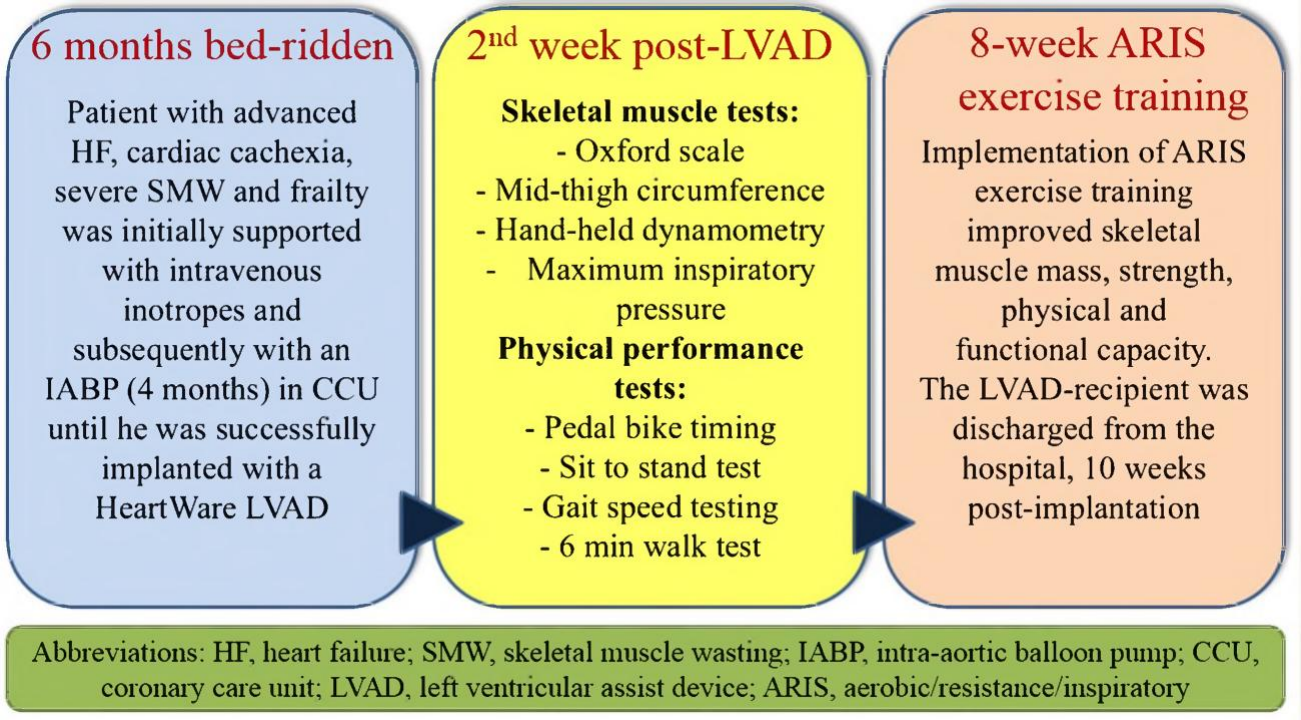


## Case presentation

A 60-year-old man with end-stage biventricular HF due to dilated cardiomyopathy was bed-ridden for 6 months, initially supported by intravenous inotropes and subsequently with an intra-aortic balloon pump (IABP—4 months) for haemodynamic stabilization in the Coronary Care Unit. Patient was very weak and frail, and despite the haemodynamic, nutritional (feeding tube for 4 months, 2000–2500 kcal/day), and physical therapy support, he developed cardiac cachexia. His body mass index (BMI) was 18.8 kg/m^2^, while he demonstrated abnormal haemoglobin (8.9 g/dL) (normal range: 14.0–17.5 g/dL), serum albumin (2.5 g/dL) (normal range: 3.5–5.5 g/dL), and C-reactive protein levels (7.8 mg/dL) (normal level < 0.9 mg/dL) (indicative values during the last week pre-LVAD implantation).^6,7^ It was very encouraging, however, that he was maintaining a good cognitive function. Interestingly, an IABP associated gradual improvement in his right ventricular function was recorded that is known to facilitate successful LVAD implantation.^8^ Detailed evaluation by our astute cardiologists indicated that his cachexia and frailty were resulting directly from HF severity and, thus, could be potentially reversible with LVAD (LVAD-responsive frailty).^9^ These ascertainments enabled the surgical team to overcome their initial reluctance for mechanical circulatory support (MCS) with an LVAD. Considering that the patient was stable enough (dependent stability) to survive the operation, he was successfully implanted with a HeartWare (Medtronic) as a bridge to heart transplantation (HTx).

**Figure 1:**
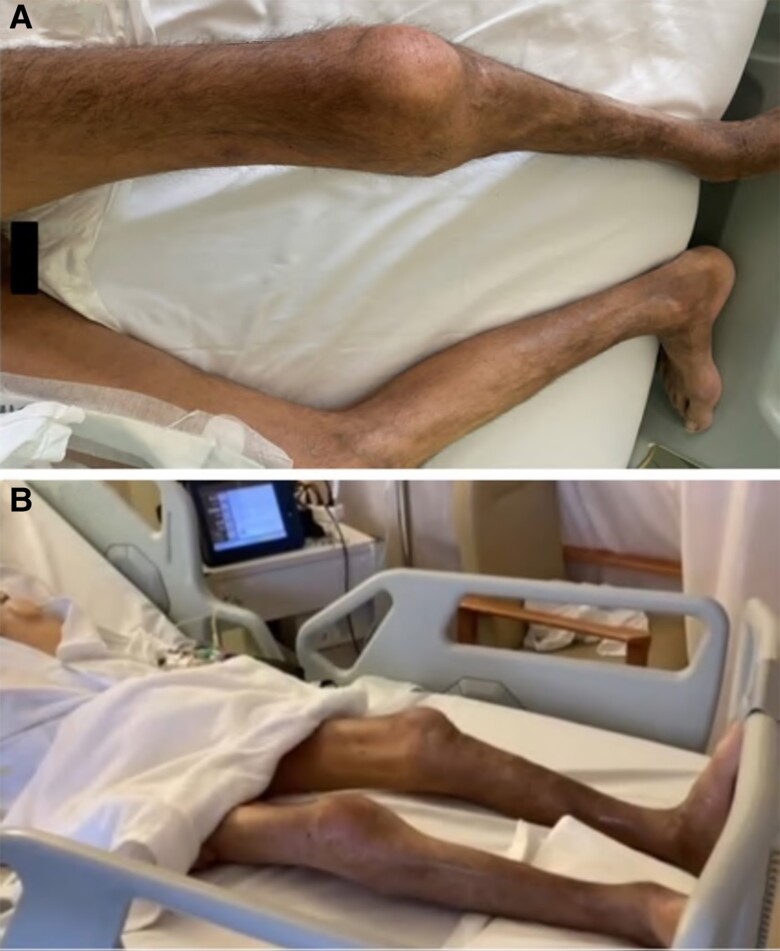
The ‘LVAD—skeleton man’ presenting with severe skeletal muscle wasting, (*A*) pre-LVAD in the Coronary Care Unit, (*B*) two weeks post-LVAD on the ward.

Two weeks post-surgery patient was transferred from the ITU to the ward. He remained frail, characterized by severe SMW, attributed to a hyper-catabolic state associated with advanced HF, bed restriction, and immobility, giving him the picture of a ‘skeleton man’ (*Figure 1A* and *B*).

He was unable to move in bed or sit without significant assistance while he was totally incapable to stand from a sitting position. He was assessed for right quadriceps muscle strength using the Oxford scale (OS) (0–5) and the quadriceps isometric peak muscle torque using a hand-held dynamometer (HHD) at mid-range. Right quadriceps muscle mass was evaluated as mid-thigh circumference (MTC) using a measuring tape. Inspiratory muscle strength was tested via maximum inspiratory pressure (PI_max_) using an electronic pressure manometer at residual volume. Physical performance battery (PPB) tests were also performed and included bed-mobility and transfers in and out of bed. Patient was mobilized in a chair with assistance, and the time of pedalling a small stationary bike at a moderate intensity (12–14) according to Borg scale (6–20) was also recorded. At a later stage, the sit to stand test (STST) was used to record number of stands in 30 s, the 4 meter gait speed testing (GST), and the 6 min walk test (6MWT) to assess exercise capacity through walking distance.^9^

**Figure 2:**
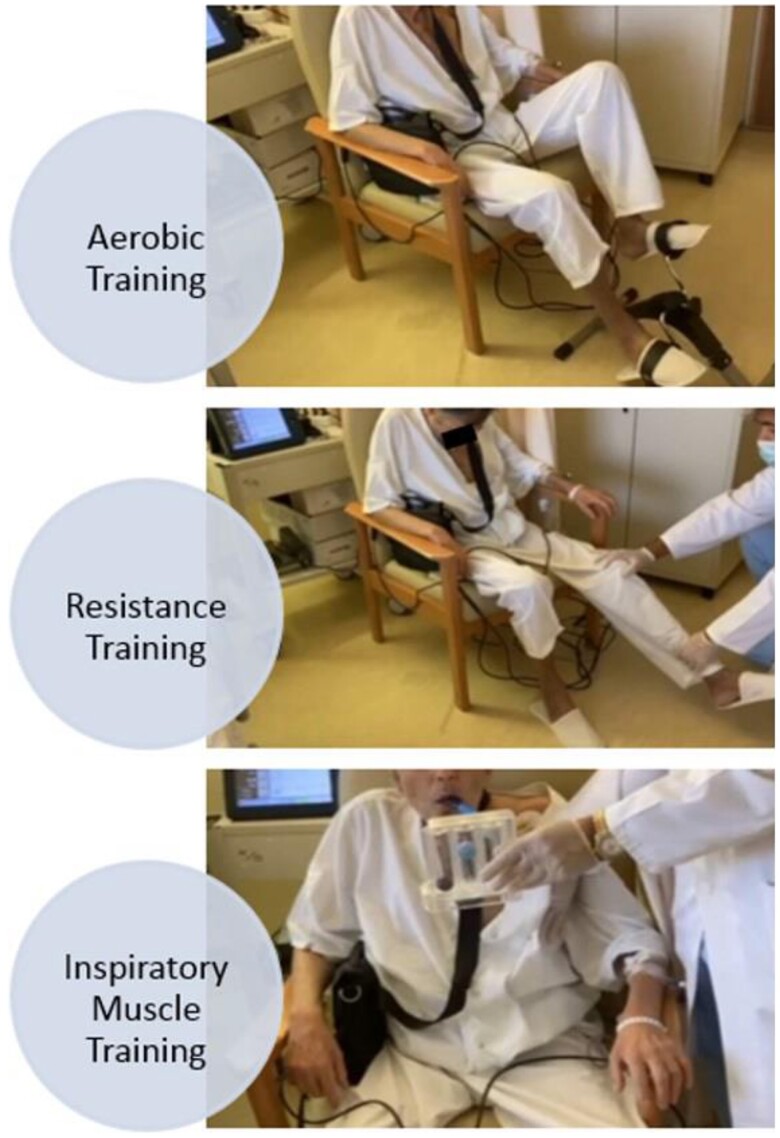
Implementation of the ARIS (aerobic/resistance/inspiratory) exercise training programme.

**Figure 3:**
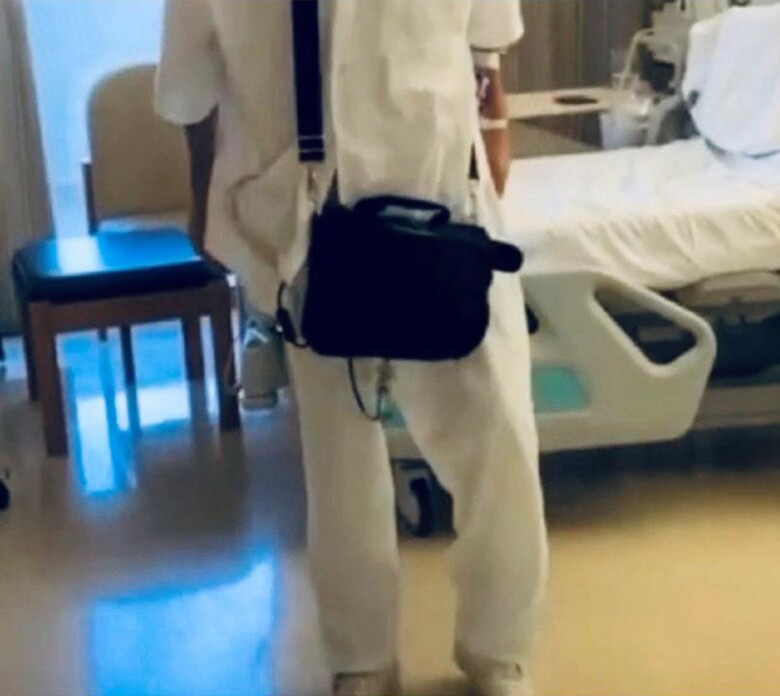
Our patient walking independent on the ward, at the end of the ARIS rehabilitation programme.

On top of routine physiotherapy (respiratory/postural/functional exercises), a structured modified aerobic/resistance/inspiratory (ARIS) exercise training (ET) programme was initiated, previously shown to offer maximal exercise benefits in HF patients and in a VAD recipient.^10,11^ Heart rate, arterial pressure, vital signs, self-reported symptoms, and LVAD function were monitored throughout exercise sessions. The programme was adjusted according to his needs and capabilities with initial emphasis given to resistance training in order to gain skeletal muscle mass and strength. A hands-on approach with emphasis on the upper and lower limbs and trunk muscle training was used that gradually progressed to the use of light weights and equipment for lower limb strengthening while upper limb resistance was delayed to ensure sternal healing. Inspiratory muscle training was performed using deep breathing exercises and an incentive spirometer (IS). Progressive resistance to inspiration was achieved by changing the angle of the IS and by decreasing the diameter of the inspiratory orifice in order to increase difficulty during inspiratory efforts and improve diaphragmatic function. Aerobic training was performed using the small stationary pedal bike with the patient progressing from a few minutes to longer training sessions (*Figure 2*). The ARIS programme lasted 8 weeks, starting with ∼30 min twice a day and then progressed to one session of 60 min daily, at a moderate exercise intensity (Borg scale: 12–14) while the patient was allowed to rest during the training sessions. Medication and LVAD parameters were constantly monitored and adjusted according to patient progress and status by a specialized cardiologist from the HF/MCS/HTx Unit, while patient was on optimum nutritional support (gastrostomy tube for 3 weeks, post-LVAD, 2000–2500 kcal/day, progressing to per os). Results of patient evaluation pre-ARIS and post-ARIS are shown in *Table 1*. At the end of ARIS training, patient was significantly stronger, walking independent on the ward, able to climb stairs with rest, and perform basic daily life activities, while his BMI was now 23.6 kg/m^2^ (*Figure 3*). He was discharged from the hospital and continued a monitored home based-ET programme while one year later, he was successfully transplanted.

**Table 1:** Results of patient evaluation pre- and post - ARIS training

	Pre-ARIS	Post-ARIS
Oxford scale (0–5)	−3	−5
MTC (cm)	25.5	31
HHD/PMT (N·m/kg)	0.67	2.01
PI_max_ (cmH_2_0)	38	59
PBT (min)	2	20
STST (reps)	UTP	5
GST (m/s)	UTP	0.571
6MWT (m)	UTP	152

ARIS, aerobic/resistance/inspiratory training; MTC, mid-thigh circumference; HHD/PMT, hand-held dynamometry/(quadriceps) peak muscle torque; PI_max_, maximum inspiratory pressure; PBT, pedal bike timing; STST, (30 s) sit to stand test; GST, (4 m) gait speed testing; 6MWT, 6 min walk test; UTP, unable to perform.

## Discussion

Early identification of sarcopenia and cardiac cachexia with treatment of malnutrition and implementation of ET are shown to be associated with better outcomes in HF patients.^3,7^ In the present case report, we demonstrate the use of simple tools for skeletal muscle and physical performance evaluation and we also show for the first time how to implement the ARIS training programme in a HF patient with severe SMW and cardiac cachexia at a very early stage post-LVAD.

Skeletal muscle wasting in end-stage HF is increasingly recognized as a biomarker of frailty and poor outcomes.^3,7,9^ A possible explanation previously suggested is the association of SMW with the inflammatory pathway that may aggravate the response to physical stresses such as cerebrovascular events and infections, and thus, patients with SMW may have difficulty in tolerating these complications, resulting in higher mortality and hospitalizations.^4^ However, despite the significance and the high prevalence of SMW (52%) in HF patients at LVAD implantation, it remains clinically under-recognized, mainly attributed to the lack of validated and easily applied diagnostic tools.^6^ Thus, simple tools such as the OS, MTC, HHD, PI_max_, and PPB tests used in the present report could be tested in a large cohort of LVAD recipients to evaluate SMW and physical performance. These tools are shown to be valid and reliable, especially when utilized in a combined manner, while are cheap, time-efficient, and easy to perform.^6,12^

Implementation of ARIS ET is highly recommended by the European Society of Cardiology guidelines for patients undergoing MCS and was associated with a spectacular improvement in our patient’s clinical profile.^13^ Patient was very co-operative, responded well to the modified ARIS ET programme, and managed to get out of his long-term hospital hardship (with him, often describing it as: ‘a journey out of hell’). The ARISTOS-HF randomized controlled trial (RCT) demonstrated that ARIS training was superior in improving limb and inspiratory muscle function, exercise capacity, and left ventricular indices, compared to different ET programmes. In addition, it was the most preferred by the HF patients.^10^ It appears that when patients engage in ET that prefer and enjoy, they perform better and improve more.^14^

However, while there is an increasing number of studies reporting the benefits of ET in patients with moderate physical capacity post-LVAD, ET RCT in very weak patients, pre- and post-LVAD implantation, is lacking.^13^ The anti-inflammatory effects of ET and its potential to reduce cytokine expression and to increase anti-apoptotic factors in HF patients are well recognized.^15^ Taking into account the association of SMW with morbidity and mortality in LVAD patients,^4–6^ initiation of very early targeted ET, even pre-LVAD, could speed up the muscle and the physical recovery in patients with sarcopenia and cardiac cachexia.^6^ In this way, early identification and clinical management of SMW could also possibly contribute to a better decision making regarding the ideal time for LVAD implantation and patient prognosis.

## Lead author biography


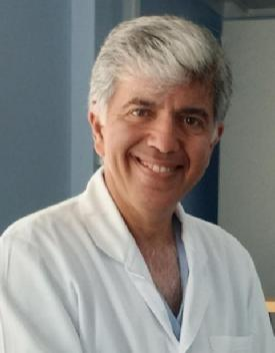


Dr Ioannis D. Laoutaris, M.Med.Sc, PhD, F.E.S.C., is a clinical/research physiotherapist graduated from University of Birmingham, UK. He is working at the Onassis Cardiac Surgery Center, Athens, Greece, while he is a member of the Onassis Scholars’ Association. His scientific interests include the research, development, and implementation of innovative exercise training methods in heart failure patients and left ventricular assist device (LVAD) recipients. The European Society of Cardiology has awarded him with the prize ‘In Recognition of Outstanding Scientific Work’ for his research activities with LVAD patients.

## Data Availability

The authors confirm that the data supporting the findings of this case report are available within the manuscript. Further details can be requested by contacting the corresponding author.
